# Biotransformation of Reactive Red 141 by **Paenibacillus* terrigena* KKW2-005 and Examination of Product Toxicity

**DOI:** 10.4014/jmb.2104.04041

**Published:** 2021-06-01

**Authors:** Chalermwoot Sompark, Jirada Singkhonrat, Niramol Sakkayawong

**Affiliations:** 1 Department of Biotechnology, Faculty of Science and Technology, Thammasat University, Rangsit Centre, Khlong Nueng, Klong Luang, Pathum Thani, Thailand, 12120; 2 Department of Chemistry, Faculty of Science and Technology, Thammasat University, Rangsit Centre, Khlong Nueng, Klong Luang, Pathum Thani, Thailand, 12120

**Keywords:** Biotransformation, decolorization, Reactive Red 141, HAT-RAPD, mung bean, toxicity

## Abstract

A total of 37 bacterial isolates were obtained from dye-contaminated soil samples at a textile processing factory in Nakhon Ratchasima Province, Thailand, and the potential of the isolates to decolorize and biotransform azo dye Reactive Red 141 (RR141) was investigated. The most potent bacterium was identified as **Paenibacillus* terrigena* KKW2-005, which showed the ability to decolorize 96.45% of RR141 (50 mg/l) within 20 h under static conditions at pH 8.0 and a broad temperature range of 30-40°C. The biotransformation products were analyzed by using UV–Vis spectrophotometry and Fourier-transform infrared spectroscopy. Gas chromatography-mass spectroscopy analysis revealed four metabolites generated from the reductive biodegradation, namely sodium 3-diazenylnaphthalene-1,5-disulfonate (I), sodium naphthalene-2-sufonate (II), 4-chloro-1,3,5-triazin-2-amine (III) and *N^1^*-(1,3,5-triazin-2-yl) benzene-1,4-diamine (IV). Decolorization intermediates reduced phytotoxicity as compared with the untreated dye. However, they had phytotoxicity when compared with control, probably due to naphthalene and triazine derivatives. Moreover, genotoxicity testing by high annealing temperature-random amplified polymorphic DNA technique exhibited different DNA polymorphism bands in seedlings exposed to the metabolites. They compared to the bands found in seedlings subjected to the untreated dye or distilled water. The data from this study provide evidence that the biodegradation of Reactive Red 141 by *P. terrigena* KKW2-005 was genotoxic to the DNA seedlings.

## Introduction 

Azo dyes play an important role as coloring agents in the textile, food, and pharmaceutical industries. They offer straightforward and cost-effective synthesis, stability, and a wider variety of colors than natural dyes [1-3]. Azo dyes absorb light in the visible spectrum due to their chemical structures, which are characterized by one or more azo groups (R1–N=N–R2) [4]. The azo can be substituted with benzene or naphthalene groups; they can contain many different substituents, such as chloro (–Cl), methyl (CH_3_), nitro (NO_2_), amino (NH_2_), hydroxyl (–OH) or carboxyl (–COOH), all of which increase solubility and fixation to fibers [4]. Unfortunately, during manufacturing and use, approximately 10-15% of the dye has been reported to be released into the environment [5, 6]. This wastewater is hazardous because some dyes and transformation products are mutagenic or carcinogenic [7, 8].

Most remediation approaches have focused on degradation by microorganisms, including bacteria, fungi, and algae [9, 10]. Among these, bacterial decolorization operates faster and more efficiently [10, 11]. Bacteria from the genus *Paenibacillus* are isolated from a variety of environments and are pertinent to humans, animals and plants [12]. Mostly discovered in soil as they are related to the roots of plants, these rhizobacteria support plant growth and development [13, 14]. They are used for remediation of various industrial wastes, including petroleum, textiles, pulp and paper, and other chemical industry byproducts that unintentionally or intentionally release large amounts of organic pollutant compounds or heavy metals. They can degrade contaminants in wastewater or at the site of environmental leaks, including fluoranthene, polycyclic aromatic hydrocarbons and benzo[a]anthracene [15-17].

The essentials affecting the decolorization and biodegradation of azo dye are temperature, pH, dye structure, dye concentration, level of agitation, oxygen, supplementation of different carbon and nitrogen sources, electron donor and redox mediator. All of these straightforwardly influence bacterial decolorization in the biological treatment process, making it more effective, faster, and virtually applicable. Tolerance to high pH is critical for industrial processes utilizing reactive azo dyes under alkaline conditions. The pH has a great effect on the effectiveness of dye decolorization and the optimal pH for dye removal by microorganism is regularly between 6.0 and 10.0 [9]. Additionally, temperature is also an important factor for all processes involved in microbial life and the remediation of water and soil. It has been noticed that the decolorization rate of azo dyes increases up to the optimal temperature followed by a negligible decrease in the decolourisation activity [9]. The reductive or oxidative status of the environment affects both the azo dye decolorization and degradation processes, directly and indirectly, leading the microbial metabolism. In the degradation of an azo dye, the reductive enzyme activities are higher under anaerobic conditions, whereas a small amount of oxygen is also required for the oxidative enzymes [9]. It has also been recognized that the transformed intermediates of azo dyes after biodegradation are exceedingly toxic and mutagenic in nature [9]. All azo dyes with a nitro group are known to be mutagenic, with high toxicity. Moreover, some azo dyes can generate toxic degradation products. The harmful derivatives are 1, 4-phenylenediamine, 1-amino-2-naphthol, benzidine, and substituted benzidines, such as o-tolidinev [18].

The toxic substances in the environment probably affect and change the organisms due to the adverse effects on the genome of the organisms, causing DNA damage. The toxicity levels of azo dyes and their transformation products were examined including phytotoxicity, ecotoxicity, genotoxicity, mutagenicity, acute toxicity, microbial toxicity, and toxicity on invertebrates [19]. Furthermore, phytotoxicity methodologies are more prevalent because they are cheaper and simpler than other methods. Due to the germination of selected seeds, the preferable species have been *Sorghum vulgare*, *Phaseolus mungo*, *Triticum aestivum*, *Sorghum bicolor*, and *Oryza sativa* [19]. The genotoxicity test is directly related to the structure and function of DNA [21]. Random amplified polymorphic DNA (RAPD) assays are polymerase chain reaction (PCR)-based techniques that improve identification of the amplified DNA regions. RAPD-PCR is considered one of the most consistent, reliable, sensitive, cost-effective and uncomplicated techniques [21]. Deletion or rearrangement of DNA base pairs may be detected without prior information about the plant genome, and only minute DNA amounts are required to estimate the degree of change in the RAPD fragments [22]. It was demonstrated that changes in RAPD profiles induced by toxic pollution exposure could also be regarded as modifications in genomic DNA template stability.

In this study we aimed to isolate and identify a bacterial species from the contaminated soil with Reactive Red 141 (RR141) dye wastewater. This RR141 dye is one of the common colorants used in various textiles, paint, garments, and related industries in Thailand. The key parameters such as pH and temperature were optimized to achieve maximum dye decolorization and biotransformation. The various intermediates formed were analyzed during the degradation of RR141 by using UV–Vis spectrophotometry, fourier-transform infrared spectroscopy (FTIR), and Gas chromatography-mass spectroscopy (GC-MS) techniques. We also determined the possible pathway in biotransformation as well as to test the phytotoxicity and genotoxicity of transformation products on Mung bean seedling. To our knowledge, this could be the first report on using the genotoxicity test by using the RAPD-PCR technique-indicated changes in genomic DNA induced by recalcitrant reactive dyes and transformation products combined with growth and changes in the plant seedling.

## Materials and Methods

### Dyes and Chemicals

**Fig. 1:**
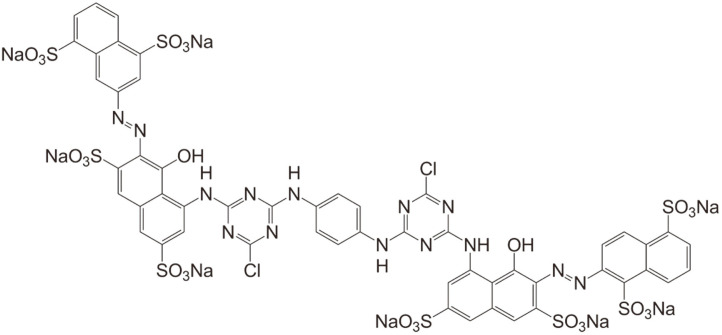
Chemical structure of diazo C.I. Reactive Red 141.

RR141 (Procion Red HE7B) is a bright red color dye, which contains two azo (-N=N-) groups. The molecular formula is C_52_H_26_C_l2_N_14_Na_8_O_26_S_8_ and the molecular weight is 1,774 g/mol. This compound has a maximum absorbance wavelength of 543 nm. The chemical structure was shown in Fig. 1. It was obtained from DyStar Thai Co., Ltd.

### Culture Medium

The primary screening medium agar for bacterial screening was nutrient agar (pH 7.0) containing 5.0 g/l peptone, 5.0 g/l NaCl, 1.5 g/l beef extract, 1.5 g/l yeast extract, 15.0 g/l agar and 20 mg/l RR141. The secondary screening medium was Bushnell and Hass basal medium [23] (pH 7.0) containing 0.2 g/l MgSO_4_, 1.0 g/l K_2_HPO_4_, 1.0 g/l KH_2_PO_4_, 0.02 g/l CaCl_2_, 0.05 g/l FeCl_3_, 0.5 g/l yeast extract, 1.0 g/l glucose, 1.0 g/l NH_4_NO_3_ and 50 mg/l RR141.

### Bacterial Screening

A total of 37 bacterial isolates were collected from dye-contaminated soil samples at a textile processing factory in Nakhon Ratchasima Province, Thailand. Our sample sites were shown in Table S1. One gram of soil was diluted with 10 ml of 0.85% (w/v) NaCl. One hundred microliters of suspension was spread on a primary screening medium agar plate which contained 20 mg/l RR141. The primary screening medium agar plates were incubated at 30°C for 48 h. All of the colonies on the agar showing clear zones around colonies were isolated and preserved at -20°C in the broth (with 15% glycerol). Primary screening produced thirty-seven isolates that decolorized the surrounding medium as shown in Table S1 and Fig. S1. The thirty-seven bacterial isolates were then subjected to secondary screening under static conditions at pH 7.0 and 30°C. A loopful of each bacterium was grown in a 250 ml Erlenmeyer flask that contained 100 ml nutrient broth. This culture was shaken at 150 rpm at 30°C until growth under exponential phase. The inoculum was diluted with nutrient broth to OD_600_ ≈ 0.582 (9 × 10^8^ colony-forming units [CFU]/ml). The inoculum was transferred to the secondary screening medium containing 50 mg/l RR141 and incubated at 30°C under static condition. One milliliter of samples was kept after 48 h, and the suspended cells were removed by centrifugation at 12,000 ×*g* for 10 min. The decolorization (%) of each bacterial strain was quantified using a UV-Vis spectrophotometer. Bushnell and Hass basal medium with RR141 dye and without inoculum was used as a negative control and culture medium without RR141 dye and without inoculum was used as blank. Based on this bacterial screening method, the bacterial isolate KKW2-005 showed the highest potential for decolorization of RR141 and was therefore selected for further studies.

### Identification of KKW2-005 through 16S rRNA Gene Amplification and Sequencing

Identification of the strain KKW2-005 was performed by sequencing the 16S rRNA gene using F984 (5′-AACGCGAAGAACCTTAC-3′) and R1378 (5′-CGGTGTGTA CAAGGCCCGGGAACG-3′) primers for amplification [24]. A bacterial cell pellet was prepared by cultivation in nutrient broth at 30°C with shaking at 150 rpm. After 24 h, the bacterial cultures were centrifuged at 12,000 ×*g* for 10 min, and the cell pellet was washed twice with peptone solution (1.0% peptone, 0.5% NaCl, pH 7.0 ± 0.2). Bacterial amplification was performed in a 20 μl reaction mixture containing 1.0 μl cell pellet, 0.5 U *Taq* DNA polymerase (Invitrogen TM Life Technologies, Brazil), 200 μM deoxynucleotides (dNTPs) and 250 nM of each of the two universal primers. Amplification included initial denaturation at 94°C for 5 min, 30 cycles of denaturation at 94°C for 30 s, annealing at 55°C for 30 s and extension at 72°C for 45 s, and a final extension at 72°C for 5 min. The reaction mixture was then examined by gel electrophoresis utilizing 1.5% (w/v) agarose. A comparison of the 16S rRNA gene sequence with those from the NCBI website (http://www.ncbi.nlm.nih.gov/) was conducted using nucleotide-nucleotide BLAST (blastn). Sequences were manually corrected and aligned with 16S rRNA gene sequences obtained from the GenBank database using the BLAST algorithm. Subsequent multiple alignments were performed using MEGA5 as described previously [25].

### Factors Affecting Decolorization

A loopful of the most significant bacterium found to efficiently decolorize RR141 in secondary screening medium was inoculated into a 250 ml Erlenmeyer flask containing 100 ml nutrient broth and shaken at 150 rpm and incubated at 30°C for 18 h. These factors were optimized to support bacterial increase during the bacterial incubation time. The inoculum was diluted with nutrient broth to OD_600_ ≈ 0.582 (9 × 10^8^ CFU/ml). The inoculum was transferred to a secondary screening medium that contained 50 mg/l RR141 and incubated at 30°C under static condition. Various pHs (5, 6, 7, 8, and 9) and temperatures (25, 30, 35, 40, and 45°C) were examined to identify the values for optimal decolorization and biodegradation. Culture media were sampled at 4-h intervals, and suspended cells were removed by centrifugation at 12,000 ×*g* for 10 min and the dry cell weight was then determined at 80°C. The supernatant was used for the decolorizing assay, with the Bushnell and Hass basal medium as a blank.

### Decolorization Assay

Absorbance measurements were performed with a UV-Vis spectrophotometer (Hitachi U-1900, Tokyo, Japan). The percentage decolorization was calculated from the difference between the initial and final absorption values of the supernatant at the maximum RR141 absorbance wavelength 543 nm. Decolorization efficiency was reported as the percentage of dye decolorization and was calculated as below [5]:

**Decolorization** (%) = [(Initial absorbance - observed absorbance)/ Initial absorbance] × 100.

(The decolorization value represented the mean of three parallel experiments).

### Metabolite Assays

After complete decolorization (20 h incubation), the decolorized medium was centrifuged at 12,000 ×*g* for 10 min, and the supernatant was used to extract metabolites with an equal volume of ethyl acetate (HPLC grade) in a separating funnel, before drying over sodium sulphate (Na_2_SO_4_). Ethyl acetate was removed using a rotary evaporator at 45°C under reduced pressure. The obtained crystals were dissolved in 1.5 ml HPLC-grade methanol and then passed through a 0.45μm cellulose acetate syringe filter (Sterlitech Corporation, USA). FTIR and GC-MS were performed to confirm the biodegradation of RR141.

Metabolites formed after RR141 decolorization were characterized by using FTIR and compared with the control dye (RR141) from 400-4,000 cm^-1^. The FTIR spectra were analyzed using OPUS software (version 7.2). GC-MS analysis was performed with a mass spectrophotometer (QP 2010 Ultra, Shimadzu). Gas chromatography was conducted in temperature-programming mode using an Agilent column (HP-5MS, length 30 m × diameter 0.25 mm × film thickness 0.25 μm). One microliter of each sample was injected in splitless injection mode with a 1.0 ml/min column flow. The initial column temperature was held at 40°C for 4 min, increased linearly at 5°C per min to 270°C and then held there for 4 min. The temperature of the injection port was 270°C, and the GC-MS interface was maintained at 275°C. Helium was used as the carrier gas at a flow rate of 1.0 ml/min and a run time of 30 min. The metabolites were identified based on the mass spectra from the NIST library (version 2.6).

### Toxicity Study

**Phytotoxicity test.** Mung bean seedlings (cultivar, Chia Tai Co., Ltd., Thailand) were used as the plant model. This plant model has been widely used in physiological and molecular toxicological examination [26]. Seeds were surface sanitized with 75% (v/v) ethanol for 5 min, then treated with 10% (w/v) sodium hypochlorite for 10 min. They were washed with tap water, then soaked for 24 h in distilled water at 25°C. The seeds were germinated to primary roots 2-4 mm in length. This procedure was done in a darkened sterile glass jar that contained three layers of No. 1 Whatman filter paper for 48 h at 25 ± 2°C. Ten germinated seeds were selected and transferred to 50.0 ± 2.0 g paddy soil pots (7.0 cm diameter and 10.5 cm height). Five pots were used for each condition. The first group was treated with 0.5 g/l of RR141 (RR1-5), while the second group was exposed to 0.5 g/l of the metabolites dissolved in sterile distilled water (KKW1-5). The control group (CT1-5) and both treated groups were tested with the same volume as 20 ml/day for 14 days. The plants were grown in a growth chamber (Contherm Phytotron) at 27 ± 2°C under a 12 h light/12 h dark photoperiod. Light intensity and relative humidity were controlled at 180 μE and 60%, respectively. Germination (%), and length of shoot and root were recorded after 14 days of germination.

**Genotoxicity using HAT-RAPD analysis.** Within each of the three groups, a single seedling was taken from five pots. Total genomic DNA of each seedling was extracted using a previously described procedure [27], with minor modifications. Briefly, 1.5 g of shoots and first leaves were ground in liquid nitrogen and mixed with 1 ml CTAB extraction buffer (2% hexadecyl trimethyl-ammonium bromide, 100 mM Tris–HCl [pH 8.0], 20 mM ethylenediaminetetraacetic acid [EDTA; pH 8.0], 1.4 M NaCl, 1% PVP-40 and 0.4% 2-mercaptoethanol). The extract was mixed with an equal volume of phenol–chloroform–isoamyl alcohol (25:24:1) and centrifuged at 12,000 ×*g* for 15 min. The mixture was then incubated at 60°C for 30 min. The supernatant was re-extracted with an equal volume of chloroform and isopropyl alcohol (24:1). After centrifugation at 12,000 ×*g*, the supernatant was transferred into a new tube and digested with RNase A at 60°C for 10 min. DNA was then precipitated by adding 0.5 ml isopropanol, and the DNA pellet was carefully washed with 70% (v/v) ethanol and dried. It was dissolved in Tris-acetate-EDTA (TAE; 40 mM Tris, 20 mM acetic acid and 1 mM EDTA) buffer and used for PCR amplification. The DNA yield was calculated by UV-Vis spectrophotometry (Shimadzu UV-1601, Australia) at 260 nm. The extracts were divided into several aliquots and stored at -20°C until use.

PCR was performed in a 20 μl reaction that contained approximately 100 ng genomic DNA, 250 nM of each primer, 200 uM each of dATP, dCTP, dGTP and dTTP, 1X reaction buffer (500 mM KCl, 15 mM MgCl_2_, 100 mM Tris-HCl pH 8.3, 1 mg/ml BSA and100 mM (NH_4_)_2_SO_4_) and 1 U *Taq* DNA polymerase (Vivantis Technologies, Malaysia). An initial screening of 72 RAPD primers was performed to test amplification profiles for polymorphisms, readability and reproducibility (primer sequences as shown in Table S4). DNA amplification reactions were performed in a thermal cycler (Biometra, Germany) using the following cycling program: 94°C for 3 min (initial denaturing step), 40 cycles of 94°C for 45 s (denaturing), 46°C for 45 s (annealing) and 72°C for 1 min (extension) and one cycle of 72°C for 8 min (final extension step). Negative controls where water replaced the template DNA were always included to monitor for contamination. After amplification, RAPD products were resolved on 1.5% agarose gels in 1X TAE buffer. The VC 100 bp Plus DNA Ladder (Vivantis) was used as the molecular size DNA standard. Ethidium-bromide-stained DNA bands were visualized using a UV transilluminator. At least two PCR amplifications were performed for each sample with RAPD primers to evaluate the reproducibility of the obtained bands. The size of each amplification product was automatically estimated using the SmartView Pro Imager System (Major Science, USA).

**HAT-RAPD profiles and data analysis.** All reactions were performed twice to verify the reproducibility of all polymorphic bands in the laboratory. HAT-RAPD data (binary matrix) was fed into NTSYS version 2.01e software to construct the dendrogram. Each HAT-RAPD profile was analyzed by scoring the presence of band as 1 and absence of band as 0, and the scores were entered into a binary matrix. Faint and unclear bands were not considered for further analysis. Genetic similarity based on Jaccard’s coefficient (JSC) was calculated among all possible pairs of the samples using the SIMQUAL module and arranged in a similarity matrix. A principal component analysis (PCA) was performed by analyzing a single set of eigenvectors to a series of variance-covariance matrices fitting.

### Statistical Analysis

Data were analyzed by one-way analysis of variance (ANOVA) with Duncan’s multiple range analysis. Readings were considered significant at *p* ≤ 0.05. The results are presented as the mean ± SD.

## Results and Discussion

### Screening of RR141-Degrading Bacteria

Thirty-seven isolates of bacteria were isolated from three sites of the contaminated soils near a textile processing factory in Nakhon Ratchasima Province, Thailand. Preliminary screening for decolorization on the agar plate supplement with 20 mg/l RR141 revealed clear zones around colonies as shown in Fig. S1. All of the selected bacteria were then subjected to secondary screening on Bushnell and Hass basal medium containing 50 mg/l of RR141 dye and incubated at 30°C for 48 h as shown in Table S2. The isolate KKW2-005 successfully decolorized RR141 (50 mg/l) with 100.00% under static conditions for 48 h as shown in Fig. S2.

### Characterization and Identification of Isolate KKW2-005

**Fig. 2:**
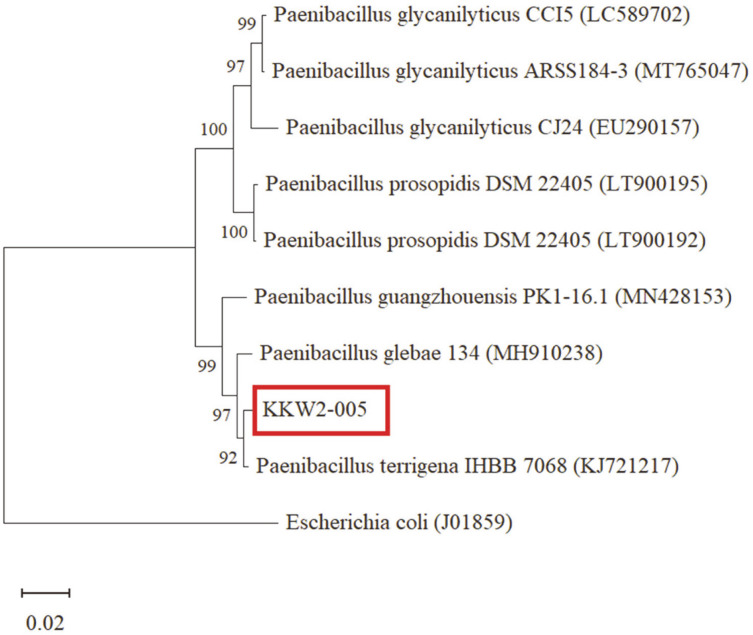
Phylogenetic tree based on 16S rRNA gene sequence (1,403 bp) comparisons that shows the relationship among members of the genus *Paenibacillus* and isolate KKW2-005. *Escherichia coli* was been taken as the out group. The sequences were retrieved from the NCBI database, and the tree was drawn using the neighbour-joining method in MEGA 5 software. The bar represents distance values calculated in MEGA, and values at nodes represent the percentage of 1,000 bootstrap replicates.

The microscopic tests revealed that isolate KKW2-005 forms circular, flat, smooth and white colonies. The cells were gram-positive and rod-shaped. The partial nucleotide genomic sequence (16S rRNA gene) of isolate KKW2-005 was used to construct the phylogenetic tree by using MEGA5 software through the neighbor-joining method and as shown in Fig. 2. The isolate KKW2-005 is closely (~99%) related to *Paenibacillus terrigena* IHBB 7068 (KJ721217). The nucleotide sequence was submitted to the National Centre for Biotechnology Information (NCBI) gene bank with accession number KF913246 and named as *P. terrigena* KKW2-005.

### Factors Affecting Decolorization

**Fig. 3:**
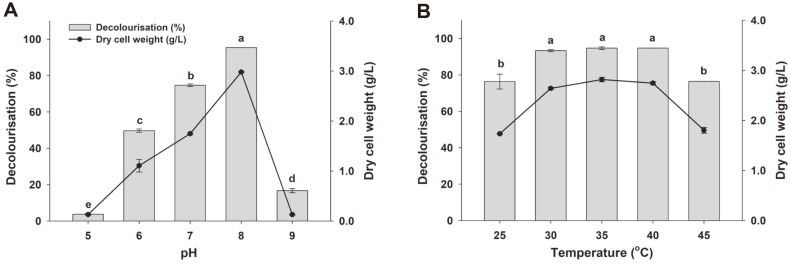
The effects of pH (A) and temperature (B) on Reactive Red 141 decolorization (%) and dry cell weight (g/l) by *Peanibacillus terrigena* KKW2-005 after 20 h. The values are shown as mean ± SD (*n* = 3). The bars marked with the same letter are not significantly different from one another based on Duncan’s multiple range analysis.

The different physicochemical parameters were studied for their effect on RR141 decolorization (Fig. 3). The effects of pH (5.0-9.0) in the BHM medium with 50 mg/l RR141 on decolorization were presented in Fig. 3A. The highest degradation (95.41 ± 0.02%) was at pH 8.0, while at pH 7.0, 6.0, 9.0 and 5.0, degradation was markedly less effective: 74.70 ± 0.68%, 49.72 ± 0.93%, 16.71 ± 1.16% and 3.77 ± 0.32%, respectively. Medium pH is an important factor regarding decolorization, and the optimal pH for color removal is often between 5.0 and 10.0 [28]. The rate of decolorization is higher at the optimum pH and tends to decrease rapidly at a strongly acid or strongly alkaline pH.

The effect of temperature on RR141 decolorization by *P. terrigena* KKW2-005 was examined from 30-45°C at pH 8.0 (Fig. 3B). The most suitable temperatures were 30, 35 and 40°C, with 93.37 ± 0.61%, 94.71 ± 0.69% and 96.45 ± 0.04% decolorization, respectively. At 25 and 45°C, this process was less favorable, with 76.34 ± 4.05% and 76.45 ± 0.03% decolorization, respectively. This performance reduction was likely due to reduced cell viability, a lower reproduction rate, and/or loss of enzyme activities [5, 6]. The decolorization of azo dyes increases up to the optimal temperature, and afterwards a marginal reduction in the decolorization activity was observed as shown in Fig. 3B. This decline in activity at higher temperatures can be attributed to the loss of cell viability or denaturing of the biotransformation enzymes [5]. The maximum rate of decolorization is generally related to optimum cell growth and physicochemical parameters which are remarkably dependent on pH and temperature for each microbial species [9, 19]. Our research showed that the *P. terrigena* KKW2-005 growth was maximum at pH 8.0 (2.98 g/l) and the temperature range was from 30 to 40°C (2.64-2.82 g/l) as shown in Fig. 3. The bacterial cell growth was directly related to the decolorization rate and coincided with the optimum physicochemical conditions [9, 10].

### Metabolites and Mechanism

**Fig. 4:**
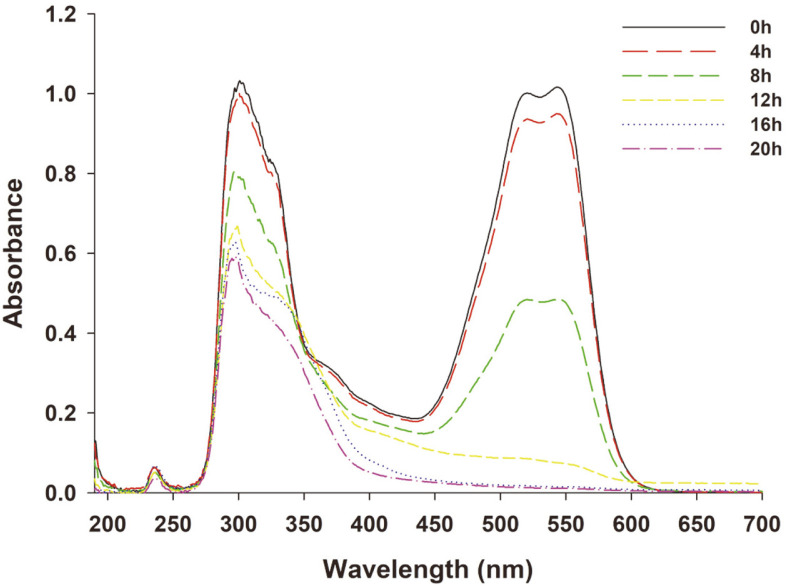
UV-Vis spectra of Reactive Red 141 and its transformation products by using *Peanibacillus terrigena* KKW2-005.

The UV-Vis spectra (190-700 nm) of the RR141 before and after treatment is shown in Fig. 4. The UV-Vis spectrum of RR141 showed a maximum wavelength at 301 nm and 543 nm. The RR141-treated dye revealed a significant decrease at the visible range near zero and disappearance of the visible range, while at UV range it also decreased from absorbance 1.0 to 0.6 after treatment by *P. terrigena* KKW2-005. This implied that RR141 was degraded by *P. terrigena* KKW2-005 under static condition.

**Fig. 5:**
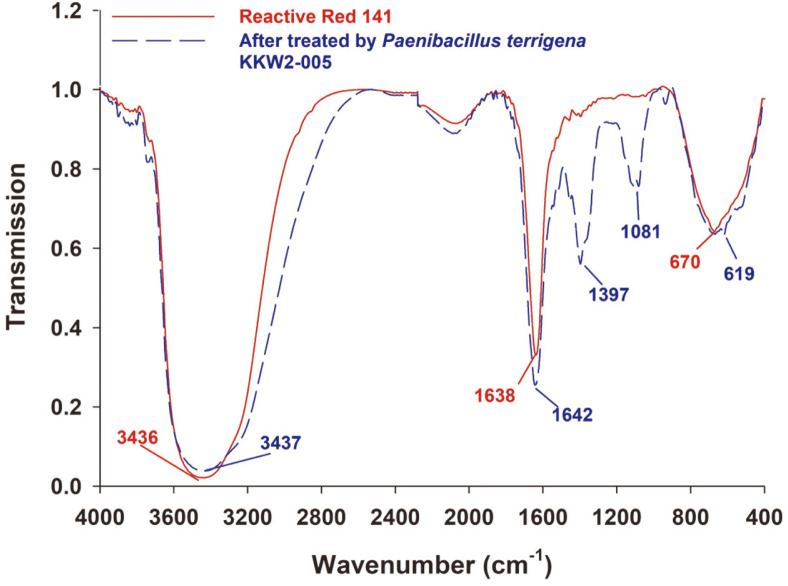
FTIR spectra of Reactive Red 141 and its transformation products by using *Peanibacillus terrigena* KKW2-005 at 20 h of incubation time.

The FTIR spectra of RR141 and its transformation products from *P. terrigena* KKW2-005 treatment are presented in Fig. 5. The RR141 dye FTIR spectrum exhibited specific peaks at 670 cm^-1^ (C-Cl, halogens), 1,638 cm^-1^ (-N=N-, azo), and 3,436 cm^-1^ (-NH, amines) [29, 30]. The metabolite FTIR spectrum showed similarity and revealed 2 more peaks at 1,081 cm^-1^ (C-N, aromatic amine), and 1,397 cm^-1^ (C=N, triazine moiety) [30].

**Fig. 6:**
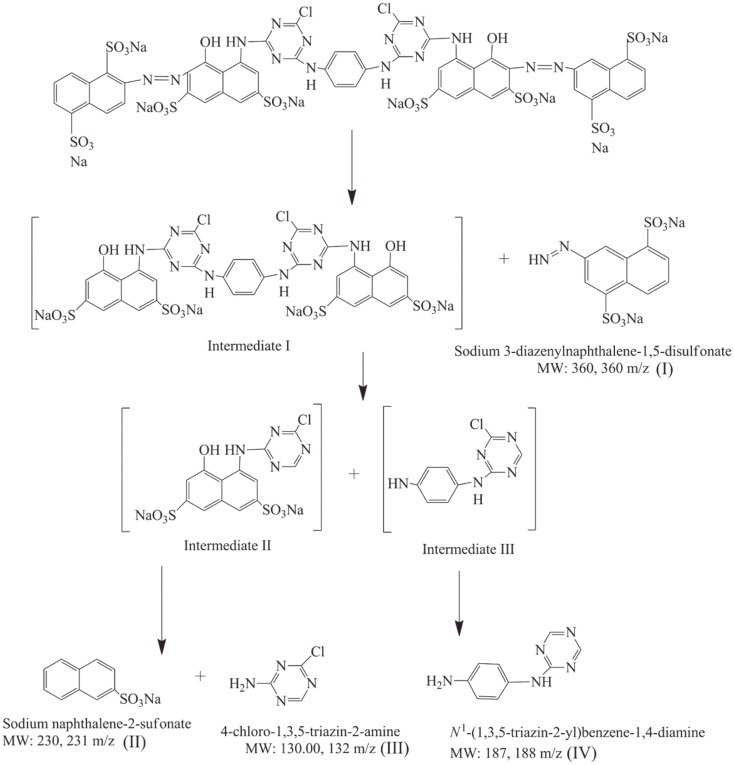
Proposed pathway of Reactive Red 141 biotransformation by *Peanibacillus terrigena* KKW2-005 via reductive cleavage.

GC-MS analysis was performed to investigate the metabolites formed during the biotransformation process (Table S3). A proposed pathway for RR141 transformation by *P. terrigena* KKW2-005 under static conditions is shown in Fig. 6. The four fragmentation patterns of reductive cleavage corresponding to RR141 transformation were observed at the retention times of 23.40, 22.79, 21.05 and 15.15 min. These fragments were assigned as 3-diazenylnaphthalene-1,5-disulfonate (I) (360 m/z), sodium naphthalene-2-sufonate (II) (230 m/z), 4-chloro-1,3,5-triazin-2-amine (III) (131 m/z) and *N^1^*-(1,3,5-triazin-2-yl) benzene-1,4-diamine (IV) (187 m/z), respectively. These data suggest that bacterial transformation involves a reductive activity rather than an oxidative pathway, in agreement with a previous report using *Rhizobium radiobacter* MTCC 8161 [29] and RR141 transformation products which mainly showed naphthalene diazonium, 1,3,5-triazine 2,4-diol, p-dinitrobenzene, and 2-nitroso naphthol. This is an indication that, in our work, bacterial degradation promoted azo group rupture, followed by the reductive cleavage of disulfonate compound, naphthenic rings and triazine rings. The identification of the fourth compound occurs by the cleavage of –N=N–C and –C–N– bonds in the proposed ways. The cleavage of the azo group generated the disulfonate and naphthalene and compounds which were further reduced to triazine and naphthalene sulfonated compounds. Triazine was reduced to 4-chloro-1,3,5-triazin-2-amine and the sulfonated compound was possibly converted to aniline, and further to ammonia, sulfide, carbon dioxide and water [31]. The disulfonate compound was likely reduced to N_2_, sulfate (SO_4_^2−^) and catechol, then further catechol mineralization through carboxylation. Apparently, non-O_2_ electron acceptors, such as sulfate, were proposed to allow subsequent anaerobic biotransformation and ultimate mineralization [32]. Decolorization under static condition may be attributable to azoreductase that degrades the azo group, having nonspecific oxidation capacity and not requiring oxygen as an electron acceptor [25].

### Toxicity Study

The phytotoxicity of RR141 and its degradation metabolites (0.5 g/l) by *P. terrigena* KKW2-005 on mung bean seedlings over 14 days are presented in Table 1 as compared to those from the control of distilled water. There was a significant decrease in the measured parameters for plants exposed to RR141 and *P. terrigena* KKW2-005-derived RR141 degradation products from distilled water. From Fig. S3, the seed germination (%), shoot length (cm), and root length (cm) for the control dye (RR141) were 62.50, 16.01 ± 3.88, and 3.72 ± 0.89, respectively. When exposed to treatment with the metabolites, these values were all higher: 75.00, 16.28 ± 3.9, and 3.91 ± 1.45, respectively. Thus, these data suggest that the metabolites from RR141 degradation were still toxic to the mung bean seedlings when compared with water. From section 3.4 metabolites and mechanism study, the transformation metabolites were assigned as sodium 3-diazenylnaphthalene-1,5-disulfonate (I), sodium naphthalene-2-sufonate (II), 4-chloro-1,3,5-triazin-2-amine (III), and *N^1^*-(1,3,5-triazin-2-yl) benzene-1,4-diamine (IV) (187 m/z), respectively. The metabolites had naphthalene and triazine as major structures. The plant could be applied to naphthalene for growth whereas it could not use triazine. The mung bean seedlings could develop in naphthalene-contaminated soil without signs of phytotoxicity and be able to remove and transfer the pollutant from the soil to the leaves. In the experiments, the seedling had the same size as the control seedling for every concentration of naphthalene applied. However, the existent of naphthalene critically affected root development, which was indirect contact with pollutants [33]. Triazine chemicals are inhibitors of photosynthesis and efficient for the selective control of grassy weeds. They have been applied as herbicides worldwide. A group of 1,3,5-triazines is the most common type of triazine herbicides [34]. This herbicide type can kill the plant by binding to quinine-binding protein in the photosynthesis II system and inhibit the electron transport in the photosynthesis process. That causes plants to turn yellow and die later [35].

**Fig. 7:**
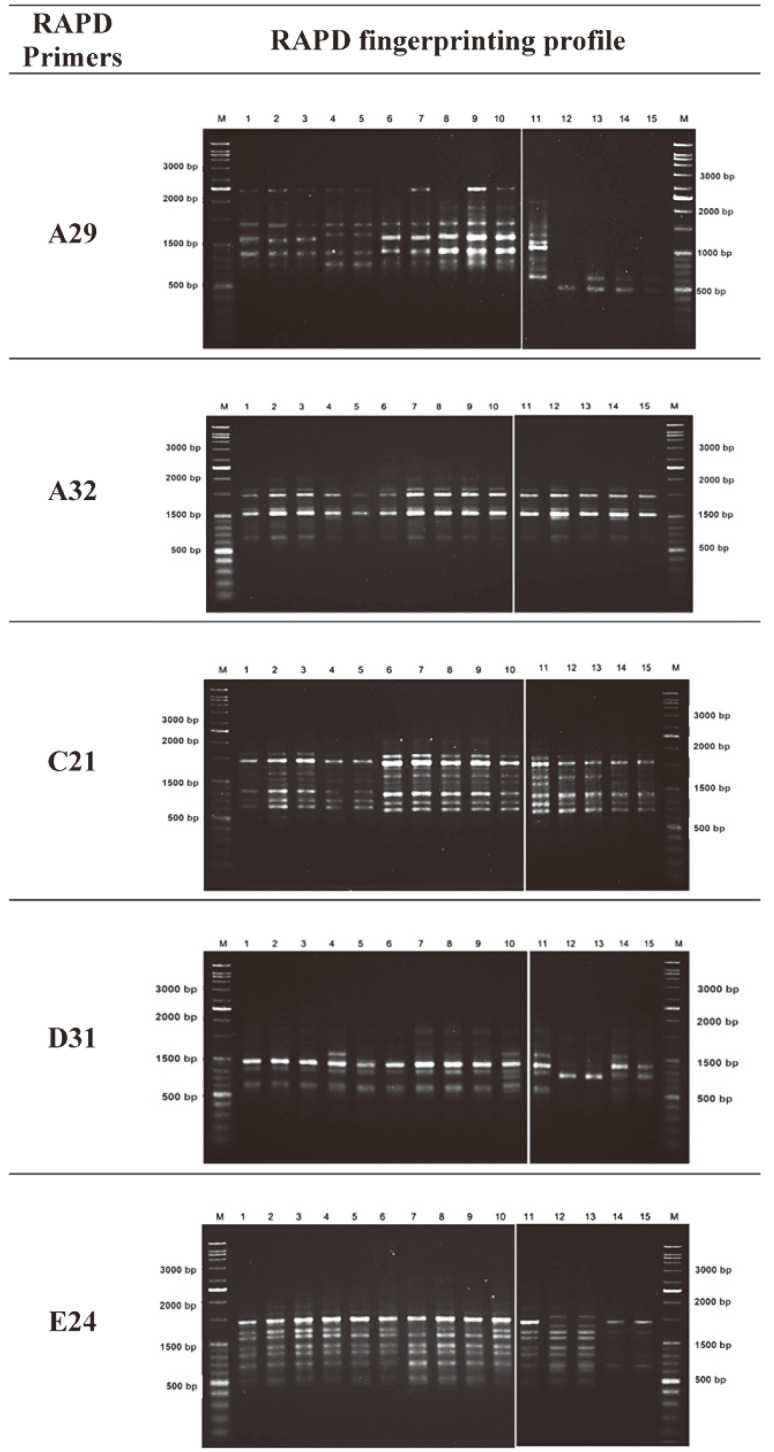
HAT-RAPD profile generated using genomic DNA extracted from mung bean seedlings exposed to Reactive Red 141 dye (untreated dye), transformation metabolites (treated dye) and distilled water (control) over 14 days.

The HAT-RAPD technique was used to examine amplified fragments after treating mung beans with the untreated dye (RR141), metabolised substances from *P. terrigena* KKW2-005 transformation, and distilled water (control). The increasing annealing temperature of the PCR technique improved the low specificity of the primers binding with the DNA template and enhanced the differentiation between closely related and morphologically indistinct samples [36]. Overall, there were myriad amplified fragments that varied from 300 to 2,300 base pairs (bp) in length (Fig. 7).

**Fig. 8:**
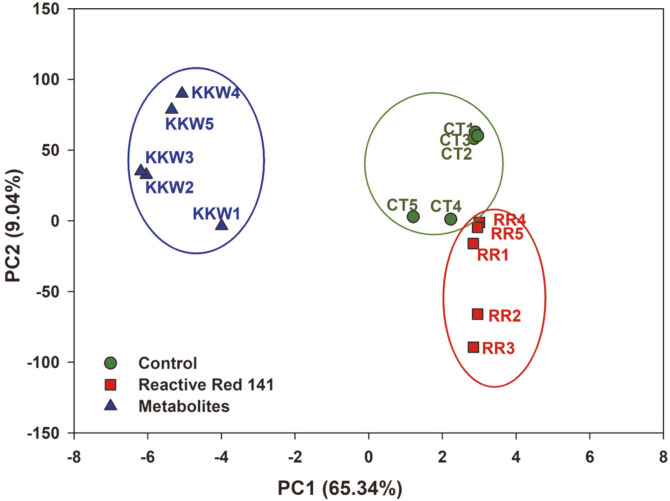
Cluster analysis of mung bean (Vigna radiata (L.) Wilczek) seedlings. Mung bean seedlings were exposed to 0.5 g/l Reactive Red 141 (RR), 0.5 g/l metabolites (KKW) and distilled water (CT) for 14 days.

The results showed five RAPD primers A29, A32, C21, D31, E24 could generate a wide array of strong and weak bands of DNA polymorphism. These studies have demonstrated both the non-presence and the presence of the bands in the DNA of mung bean seedlings exposed to RR141 and metabolized substances from *P. terrigena* KKW2-005 degradation compared to those of controls. The difference in DNA fingerprinting profiles (non-presence and the presence of the bands) might be due to the changing or damage in oligonucleotide primer sites due to genomic rearrangement that occurred and lead to DNA damage and point mutations in the plant genome [21]. Principle component analysis could discriminate the genotypes into clusters in a PCA plot. This data indicated substantial genetic diversity among mung bean seedling DNA as shown in Fig. 8.

The genetic diversity among mung bean seedlings of the RR141-treated seedling subgroup was similar to those of distilled water-treated seedlings by 65.34% PC1 and 9.04 % PC2. The metabolite-treated seedling subgroup (KKW1-5) was far from both groups of the RR141- (RR1-5) and distilled water-treated seedlings (CT1-5). These results suggested that the DNA polymorphism bands in plant exposed to RR141 metabolites derived from *P. terrigena* KKW2-005 degradation had more different genotypes than the untreated dye and distilled water. This finding could be explained by the molecular mechanism of the active gradient uptake of RR141 metabolites derived from *P. terrigena* KKW2-005 transformation. These metabolite substances have more toxicity than untreated dye caused by the smaller molecules of metabolites. Mung bean could be an uptake of the smaller molecules of metabolites more easily and rapidly than those bigger molecules (RR141). The plant was unhealthy with DNA strand breaks that may have been introduced directly by genotoxic effects and then through the induction of apoptosis or necrosis interactions [37]. Even though the decolorization and biotransformation by *P. terrigena* KKW2-005 under static condition rapidly degraded RR141 to small molecules the metabolites still stimulated plant genomic DNA changes. Naphthalene showed as one of the major metabolites of this work and had more effect on plant genomic DNA change. Naphthalene, a cytotoxic moiety, is an extensively explored aromatic conjugated system with applications in various pathophysiological conditions, viz. anticancer, antimicrobial, anti-inflammatory, antiviral, antitubercular, antihypertensive, antidiabetic, anti-neurodegenerative, antipsychotic, anticonvulsant, and antidepressant. Derivatives of naphthalene were responsible for the covalent interaction with cysteine amino acids of cellular proteins and cytotoxicity [38], whereas the toxicity of triazines showed relatively low acute toxicity but exerted inhibitory effects on tadpole growth and development [34].

The naphthalene from metabolites of biotransformation could be removed by an anoxic–aerobic continuous flow combined bioreactor under a continuously oxic and anoxic system. In continuous mode, the studies were conducted, and naphthalene was increased in consecutive spike dose. A novel *Pseudomonas aeruginosa* was reckoned and injected on a vertical anoxic–aerobic continuous flow combined bioreactor. This method can be effectively applied for naphthalene biodegradation [39]. Moreover, *Pseudomonas* sp. strain ADP was the best-characterized bacterial strain capable of being used for triazine compound elimination. The major steps of the atrazine degradation pathway are hydrolysis, dealkylation, deamination and ring cleavage. The end of this process could produce cyanuric acid converted to ammonia and carbon dioxide by atrazine chlorohydrolase enzymes [35].

## Conclusions

This study demonstrated that RR141 biodegradation by *P. terrigena* KKW2-005 isolated from dye-contaminated soil in Nakorn Ratchasima, Thailand, effectively occurred under static conditions. The influence of pH and temperature was affected by biodegradation in this study. UV–Vis spectroscopy and FTIR analysis confirmed the biodegradation and a possible pathway of reductive cleavage was proposed by GC–MS analysis. The transformation metabolites were proposed as sodium 3-diazenylnaphthalene-1,5-disulfonate (I), sodium naphthalene-2-sufonate (II), 4-chloro-1,3,5-triazin-2-amine (III), and *N^1^*-(1,3,5-triazin-2-yl) benzene-1,4-diamine (IV) (187 m/z), respectively. The major structures of the final metabolites were naphthalene and triazine. Phytotoxicity studies suggested that the metabolites were still toxic more than control whereas, seedling DNA exposed to these metabolites showed different DNA polymorphism bands compared to seedlings exposed to the unaltered RR141 dye. The PCA plots for genetic similarity relationships of the degradation metabolites were still different from distilled water. Therefore, the metabolites were genotoxic. Thus, the application of *P. terrigena* KKW2-005 might not be suitable for reactive dye treatment and release to the environment. The reductive cleavage of azo bonds leads to the formation of toxic aromatic amines under anaerobic conditions, so it was still necessary to assess the extent of biotransformation after dye treatment. The sequential anaerobic-aerobic biological methods might be appropriate to eliminate naphthalene and triazine after biotransformation or chemical methods can be applied to separate those compounds for future uses.

## Supplemental Materials

Supplementary data for this paper are available on-line only at http://jmb.or.kr.

## Figures and Tables

**Table 1:** Phytotoxicity of Reactive Red 141 (RR141) and its transformation products (0.5 g/l) by *Peanibacillus terrigena* KKW2-005 on mung bean (*Vigna radiata* (L.) Wilczek) seedlings after 14-days exposure.

Parameters	Samples		

	Distilled water (Control)	RR141 (Untreated)	RR141 metabolites (Treated)
Germination (%)	100.00	62.50*	75.00**
Shoot length (cm; mean ± SD)	17.03 ± 3.41	16.01 ± 3.88*	16.28 ± 3.91**
Root length (cm; mean ± SD)	4.75 ± 1.68	3.72 ± 0.89*	3.91 ± 1.45**

*Significantly different from control (distilled water), *p* ≤ 0.05.

**Significantly different from control (distilled water), *p* ≤ 0.01.
