# Supplementary material for: Biotransformation of Reactive Red 141 by Paenibacillus terrigena KKW2-005 and Examination of Product Toxicity
Source: J Microbiol Biotechnol. 2021 Jun 1;31(7):967–77. doi: 10.4014/jmb.2104.04041 (PMC9705871; doi:10.4014/jmb.2104.04041)
Supplement: Supplementary file 1 [file jmb-31-7-967-supple.pdf]

J. Microbiol. Biotechnol.
https://doi.org/10.4014/jmb.2104.04041

Supplementary materials

Biotransformation of Reactive Red 141 by *Paenibacillus terrigena* KKW2-005 and examination of product toxicity

Table S1 Primary screening of bacteria from textile effluent contaminated soils in Nakorn Ratchasima province, Thailand.

**Sample sites (°N, °E)**	**Number of Isolates**	**Isolates**
**Site 1 (14.98, 102.09)**	5	KKW1-001, KKW2-005, KKW3-001, KKW3-002, KKW3-003
**Site 2 (14.90, 102.08)**	26	KNP-011, KNP-012, KNP-013, KNP-018, KNP-101, KNP-104, KNP-107, KNP-108, KNP-144, KNP-151, KNP-153, KNP-162, KNP-163, KNP-201, KNP-301, KNP-302, KNP-403, KNP-501, KNP-502, KNP-505, KNP-506, KNP-508, KNP-601, KNP-701, KNP-802, KNP-806
**Site 3 (15.20, 101.64)**	6	WJP1-001, WJP1-007, WJP2-002 WJP2-003, WJP2-001, WJP2-004

Table S2. Secondary screening of bacteria from soils of Nakhon Ratchasima, Thailand for 48 h under static condition.

No.	Isolates	Decolurisation (%)
1	KKW1-001	23.75±1.42
2	KKW2-005	100.00±0.00
3	KKW3-001	22.54±3.15
4	KKW3-002	1.05±0.43
5	KKW3-003	28.91±2.25
6	KNP-011	23.19±1.39
7	KNP-012	28.30±3.16
8	KNP-013	24.34±4.33
9	KNP-018	27.36±1.25
10	KNP-101	23.48±3.10
11	KNP-104	29.23±0.56
12	KNP-107	24.14±2.80
13	KNP-108	21.10±1.66
14	KNP-144	28.89±1.61
15	KNP-151	21.07±2.65
16	KNP-153	3.15±3.25
17	KNP-162	21.26±1.73
18	KNP-163	27.34±1.87
19	KNP-201	26.89±1.98
20	KNP-301	27.39±1.20
21	KNP-302	19.60±1.54
22	KNP-403	29.56±2.53
23	KNP-501	28.92±3.98
24	KNP-502	18.28±4.04

No.	Isolates	Decolurisation (%)
25	KNP-505	29.63±3.53
26	KNP-506	23.82±2.72
27	KNP-508	33.93±0.70
28	KNP-601	19.48±3.98
29	KNP-701	28.23±2.20
30	KNP-802	19.52±4.47
31	KNP-806	21.59±2.31
32	WJP1-001	1.35±1.18
33	WJP1-007	30.58±0.63
34	WJP2-002	22.57±2.47
35	WJP2-003	3.11±1.46
36	WJP2-001	32.65±0.59
37	WJP2-004	1.63±0.79

Table S3. Gas chromatography/mass spectrometry (GC/MS) spectral data of Reactive Red 141 biotransformation products by *Peanibacillus terrigena* KKW2-005

Metabolite name	RT (min)	*m/z*	Mass spectra
Sodium 3-diazenylnaphthalene-1,5-disulfonate	23.40	360	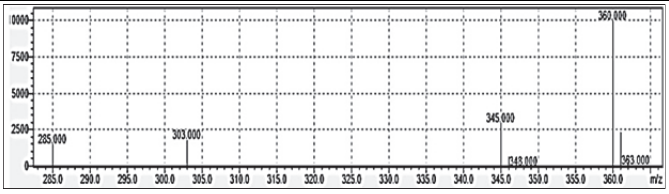
Sodium naphthalene-2-sufonate	22.79	230	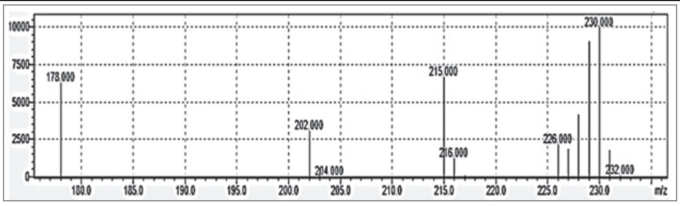
4-chloro-1,3,5-triazin-2-amine	21.05	131	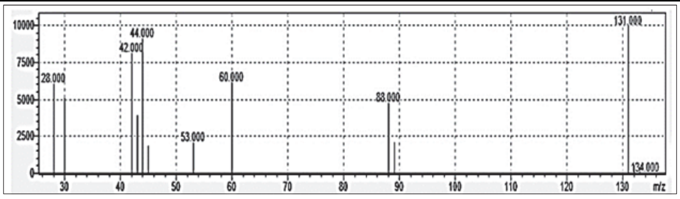
N^1-(1,3,5-triazin-2-yl) benzene-1,4-diamine	15.15	187	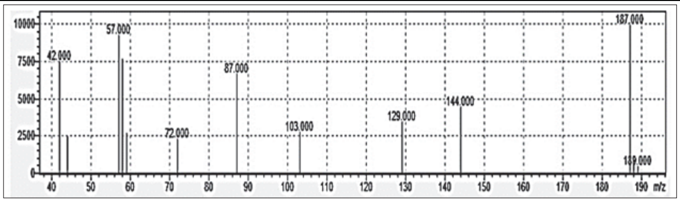

Note: RT, retention time.

Table S4. HAT-RAPD Primers

Primer	Sequences (5′-3′)	Primer	Sequences (5′-3′)	Primer	Sequences (5′-3′)
A21	AGAATTGGACGA	C21	GGAGAGCGGACG	E21	TGCTTCGTATTA
A22	GCCTGCCTCACG	C22	GGTCACCGATCC	E22	GGAATGGAACCG
A23	ACTGACCTAGTT	C23	CCGTCTTTTCTG	E23	AGGTACGCCGCA
A24	CTCCTGCTGTTG	C24	CCTTGGCATCGG	E24	CCGGAGTGGATG
A25	CTCAGCGATACG	C25	AGATTCTTACTG	E25	CCGGAGTGGATG
A26	ACTGAGAAAATA	C26	GCGTTCGAACGA	E26	CTGCCTGTACCA
A27	ATCGCGGAATAT	C27	GCATTGCAATCG	E27	CCATTGTCGGTA
A28	ATTTGGATAGGG	C28	GTCGACGCATCA	E28	CGCCCTGCAGTA
A29	GGTTCGGGAATG	C29	GTCGCCTTACCA	E29	GTTATGCAAGGG
A30	GACCTGCGATCT	C30	TATTGGGATTGG	E30	TACCTGGTTGAT
A31	AAGGCGCGAACG	C31	TCTGCTGACCGG	E31	GAGGACAGCAA
A32	TTGCCGGGACCA	C32	TCTACACGAAGT	E32	CAGGAACAGCAA
B21	AAGCCTATACCA	D21	GGCGATTCTGCA	F21	AACCTTTAGGGC
B22	GGTGACTGGTGG	D22	TGCCCACTACGG	F22	AAGAGGGTTGAC
B23	GGTGCCGGAGCA	D23	ACCATCAAACGG	F23	CCATCCGCACGA
B24	CACACTACTTAT	D24	GTGCAATTTGGC	F24	ACTGTTATAACG
B25	AGCACTGAATCT	D25	GTTTTGTCACCG	F25	CCAGATCCGAAT
B26	ATGAGAAAGGAA	D26	GATGAGCTAAAA	F26	CTCAGCATTGAT
B27	GGCGGTTATGAA	D27	AGAATGTCCGTA	F27	CAGGTGGGAGTA
B28	GTCATTAAAGCT	D28	ACTGAGGGGGGA	F28	CCAAGATCCATT
B29	GCCATCGAAAAA	D29	ATCAAGTATCCA	F29	GCCGCTAATATG
B30	CTTAGGTTACGT	D30	GAGACTACCGAA	F30	ACTTTCGCCGAA
B31	CACAAGGAACAT	D31	GGAGGTCGACCA	F31	ATCGTGACGCCG
B32	ATCGCGGCTTAT	D32	AAGCTGGGGGGA	F32	TTCAACATCGAC

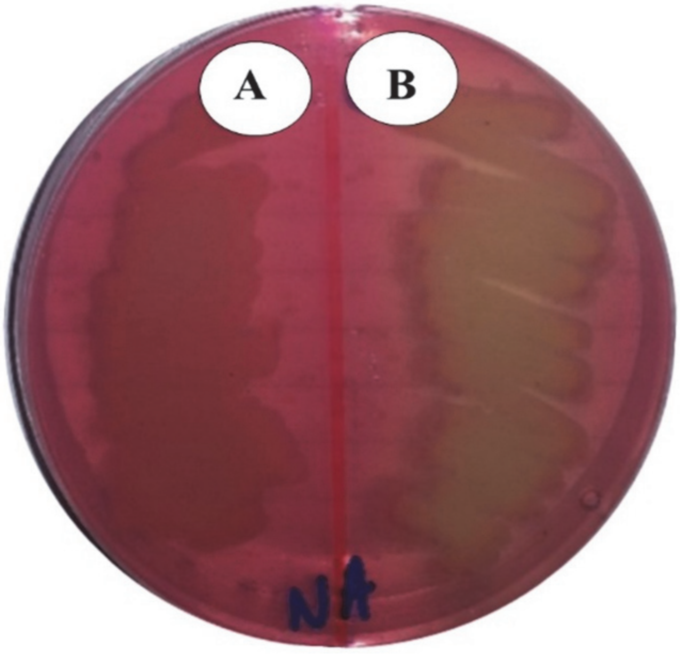

Fig. S1 Primary screening of bacteria grown on nutrient agar (NA) containing 20 mg/l of Reactive Red 141 dye and incubated at 30ºC for 48 h. The letter (A) indicate that isolate could not decolurisation of RR141; letter (B) indicate that isolate could decolurisation of RR141

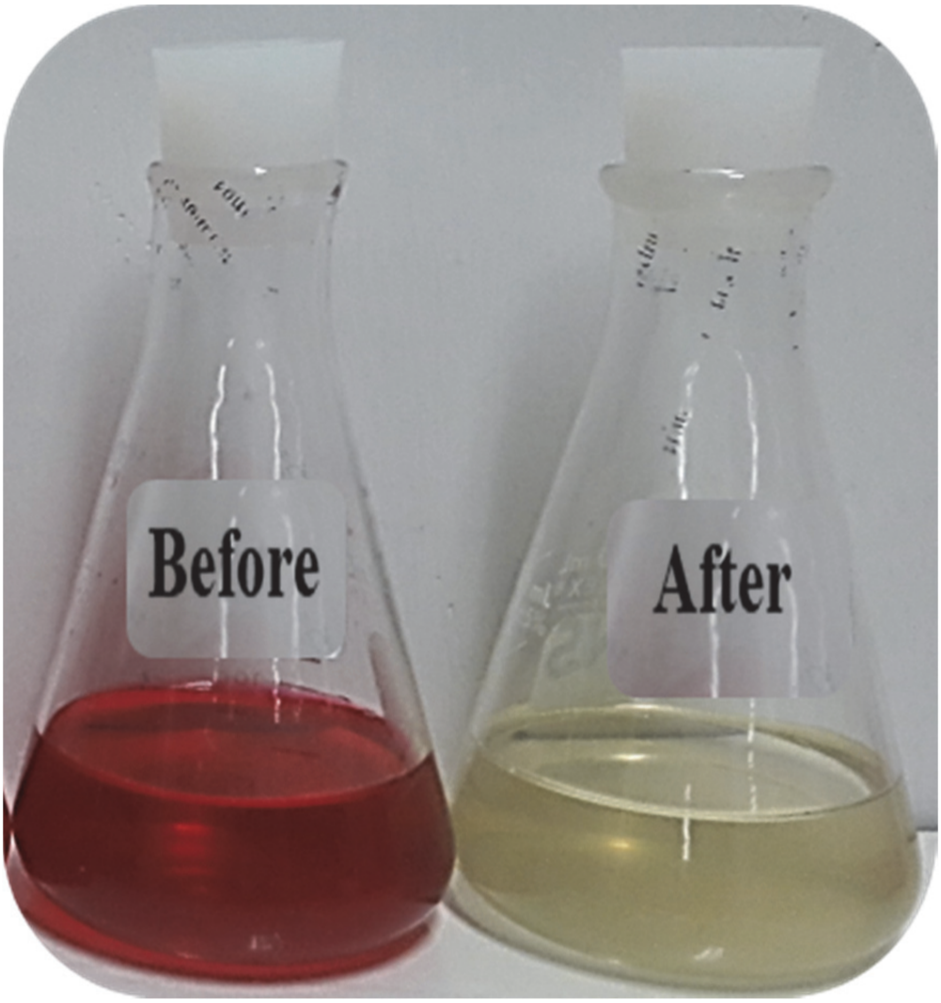

Fig. S2 *Paenibacillus terrigena* KKW2-005 grown on Bushnell and Hass basal medium containing 50 mg/l of Reactive Red 141 dye and incubated at 30ºC for 48 h.

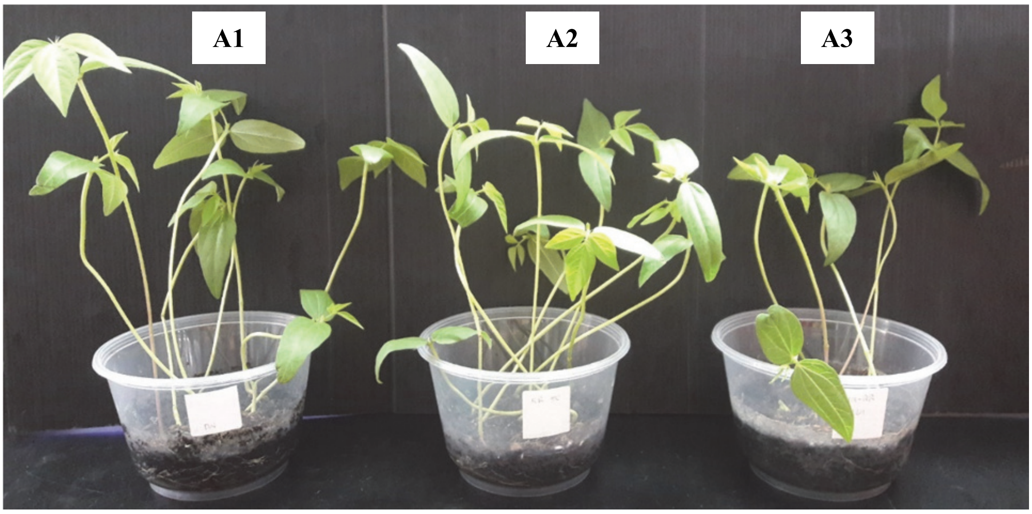

Fig. S3 Pot experiments of mung bean seedlings exposed to distilled water (A1), metabolised substances from *P. terrigena* KKW2-005 transformation (A2) and untreated RR141 dye (A3) over 14 days.
